# Supplementary material for: CRP Monitoring in Early Hospitalization: Implications for Predicting Outcomes in Patients with COVID-19
Source: Pathogens. 2023 Nov 4;12(11):1315. doi: 10.3390/pathogens12111315 (PMC10675493; doi:10.3390/pathogens12111315)
Supplement: Supplementary file 1 [file pathogens-12-01315-s001.zip › pathogens-2670515-supplementary.pdf]

pathogens

MDPI

Supplemental Figures and Tables

Table S1. Binning methods for comorbidities.

Category	Diseases Included
Arrythmia	Atrial Fibrillation, Atrial Flutter, Atrioventricular Nodal Reentry Tachycardia, AV Block, Bradycardia, Brugada Syndrome, Cardiac Arrest, Heart Block, Pacemaker or Defibrillator, Prolonged Qtc, Sick Sinus Syndrome, Ventricular Fibrillation, Ventricular Tachycardia
Coronary Artery Disease or MI History	Acute Coronary Syndrome, Arteriosclerotic Heart Disease, Coronary Artery Bypass Graft, Coronary Artery Disease, Coronary Stent Placement, Myocardial Infarction, and Percutaneous Coronary Intervention
DVT/PE	Arterial Occlusion of Brachial Artery, Deep Vein Thrombosis, Inferior Vena Cava Filter, Pulmonary Embolism
Heart Failure	Cardiomyopathy, Chronic Heart Failure, Diastolic Dysfunction, Dilated Cardiomyopathy, Heart Failure, Idiopathic Cardiomyopathy, Ischemic Cardiomyopathy, Left Ventricular Systolic Dysfunction, Non-Ischemic Cardiomyopathy, Systolic Cardiomyopathy, Viral Cardiomyopathy
Hypertension	Hypertension, Preeclampsia
Cerebrovascular Disease	Anoxic Brain Injury, Basal Ganglia Infarct, Brain Aneurysm, Cerebellar Artery Stent-Graft, Cerebellar Hemorrhage, Cerebrovascular Accident / Stroke, Chronic Infarct on Head CT, Intracerebral Aneurysms, Intracranial Hypertension, Intraparenchymal Hemorrhage, Subarachnoid Hemorrhage, Subdural Hematoma, Transient Ischemic Attack, Wallenberg's Syndrome
Genitourinary Disease	Atypical Hyperplasia Of Endometrium, Benign Prostate Hyperplasia, Bladder Implant, Cervical Implant, Chronic Pelvis Abscess, Dysfunctional Uterine Bleeding, Epididymitis / Orchitis, Fournier Gangrene, Gangrene Of the Penis, HSV Genital, Hysterectomy, Menorrhagia, Ovarian Cyst, Overactive Bladder, Penile Syringoma, Perianal Fistula, Polycystic Ovarian Syndrome, Prostatitis, Recurrent UTIs, Transurethral Resection of the Prostate, Ureteral Colic, Ureteral Strictures, Urinary Bladder Stones, Urinary Incontinence, Urinary Retention, Uterine Fibroid
GI Disease	Achalasia, Alcohol Induced Chronic Pancreatitis, Barrett's Esophagus, Candida Esophagitis/Gastritis, Cholecystectomy, Cholecystitis, Choledocholithiasis, Cholelithiasis, Chronic Constipation, Chronic Dysphagia, Colitis, Cyclic Vomiting Syndrome, Dieulafoy's Lesion Gastritis, Diverticulitis, Diverticulosis, Duodenum Ulcer, Endoscopic Retrograde Cholangiopancreatography With Sphincterotomy And Stent, Esophageal Ulcers, Esophagitis, Gallbladder Disease, Gallbladder Sludge, Gallstone Pancreatitis, Gastric Diverticulum, Gastric Ulcer, Gastritis, Gastroesophageal Reflux Disease, Gastrointestinal Bleed, Gastrointestinal Disease, Hartmann's Operation, Hemorrhoids, Hernia, Hernia Repair, Ileal Conduit, Ileostomy, Intraabdominal Abscesses, Intussusception, Irritable Bowel Syndrome, Multiple Bowel Obstructions, Pancreatic Pseudocyst, Pancreatitis, Peptic Ulcer, Peptic Ulcer Disease, Perforated Cholecystoduodenal Fistula, Perigastric Abscess, Pouchitis, Rectal Prolapse, Recurrent C. Diff. Colitis, Reflux, Reflux Esophagitis, Sigmoid Colostomy Stricture, Small Bowel Obstruction, Stercoral Ulcers, Surgery For Colon Infection, Ulcerative Colitis, Ulcerative Proctitis
Anemia	Alpha-Thalassemia Anemia, Anemia, Anemia of Chronic Disease, Anemia of Renal Disease, Autoimmune Hemolytic Anemia, Beta-Thalassemia, Beta-Thalassemia Sickle Cell Trait, Chronic Anemia, Hbsc Hemoglobinopathy, Iron Deficiency Anemia, Microcytic Anemia, Normocytic Anemia, Pernicious Anemia, Sickle Cell Disease, Thalassemia
Malignancy	Acute Lymphocytic Leukemia, Acute Myelocytic Leukemia, Adenocarcinoma, Atypical Meningioma, Basal Cell Carcinoma, B-Cell Acute Lymphocytic Leukemia, B-Cell Lymphoma, Biliary Ductal Carcinoma, Bladder Cancer, Brain Mass, Brain Tumor, Breast Cancer, Bronchogenic Carcinoma, Burkitt's Lymphoma, Calvarial Tumor Resection, Cervical Cancer, Chronic Lymphocytic Leukemia, Chronic Myeloid Leukemia, Colon Cancer, Complex Atypical Hyperplasia Of Endometrium S/P Hysterectomy, Diencephalon Tumor, Diffuse Large B-Cell Lymphoma, Endometrial Cancer, Familial Adenomatous Polyposis, Gastric Cancer, Gynecological Cancer, Hodgkin's Lymphoma, Intraductal Papillary Mucinous Neoplasm, Intrathoracic Tumor, Laryngeal Squamous Cell Carcinoma, Liver Cancer, Lung Cancer, Lung Mass S/P Chemotherapy And Radiotherapy, Lymphoma, Malignant Neoplasm Of The Tongue And Pharynx, Malignant Pleural Effusion, Melanoma, Metastatic Spinal Compression

Category	Diseases Included
	Fractures, Monoclonal Gammopathy Of Undetermined Significance, Multiple Myeloma, Myelodysplastic Syndromes, Nasopharyngeal Cancer, Non-Hodgkin's Lymphoma, Non-Small Cell Lung Cancer, Ovarian Cancer, Pancreatic Cancer, Pancreatic/Biliary Adenocarcinoma, Pancytopenia S/P Recent Chemotherapy, Papillary Thyroid Cancer, Penile Cancer In Situ, Pharyngeal Carcinoma, Prostate Cancer, Rectal Cancer, Renal Cancer, Renal Carcinoma, Renal Cell Carcinoma, Renal Mass With Resection, Sigmoid Colon Cancer Adenocarcinoma, Skin Cancer, Squamous Cell Carcinoma, Thyroid Cancer, Urothelial Cancer, Uterine Cancer, Vulvar Cancer
Diabetes	Diabetes, Diabetes Mellitus, Diabetes Mellitus Type 1 / Insulin Dependent Diabetes Mellitus, Diabetes Mellitus Type 2 / Non-Insulin Dependent Diabetes Mellitus, Diabetic Foot Ulcers, Diabetic Neuropathy, Diabetic Retinopathy, Gestational Diabetes Mellitus, New Onset Diabetes / Newly Diagnosed Diabetes Mellitus
Hyperlipidemia	Dyslipidemia, Hypercholesterolemia, Hyperlipidemia, Hypertriglyceridemia
Hypothyroidism	Hashimoto's Thyroiditis, Hypothyroidism
Musculoskeletal Disease	Arthritis, Arthroplasty, Avascular Necrosis Of Hip, Back Surgery, Bilateral Inguinal Hernia S/P Repair, Bunionectomy, Calcium Pyrophosphate Deposition Disease, Chronic Back Pain, Compression Fracture, Gout, Hemiarthroplasty, Herniation Of Nucleus Pulposus, Hip Fracture, Hip Replacement, Knee Placement, Kyphosis, Laminectomy, Lower Extremity Amputation, Lumbar Radiculopathy, Multiple Orthopedic Surgeries, Olecranon Bursitis / Cellulitis, Open Reduction And Internal Fixation, Osteoarthritis / Degenerative Joint Disease, Osteopenia, Osteoporosis, Rhabdomyolysis, Rib Fractures, Sacral Decubitus Ulcer, Sciatica, Scoliosis, Spina Bifida, Spinal Destruction, Spinal Stenosis, Spine Surgery, Spondylosis
Dementia	Alzheimer's Disease, Dementia, Lewy Body Dementia, Parkinson's Disease
Other Neurological Disease	Aphasia, Arnold-Chiari Malformation, Ataxia, Bell's Palsy, Central Canal Stenosis / Foraminal Narrowing, Cerebellar Cavernoma, Chronic Fatigue Syndrome, Encephalitis, Encephalomalacia, Encephalopathy, Essential Tremor, Fibromyalgia, Guillain Barre Syndrome, Hallervorden-Spatz Disease, Head Injury / Traumatic Brain Injury, Hydrocephalus, Idiopathic Myeloneuropathy, Korsakoff's Disease, Meningioma S/P Resection, Migraines, Multiple Sclerosis, Muscular Weakness Secondary To Polio, Neurogenic Bladder, Neurosyphilis, Paraplegia, Polio, Restless Leg Syndrome, Sacral Neuromodulatory Implant, Spinal Cord Compression, Spinal Cord Injury, Vocal Cord Paralysis
Anxiety	Generalized Anxiety Disorder
Depression	Major Depressive Disorder, Past Suicidal Ideation, and Suicide Attempt
Chronic Obstructive Pulmonary Disease (COPD)	Emphysema, Bronchitis, and Unspecified COPD
Other Pulmonary Disease	Bronchiectasis, Chronic Respiratory Failure, Fungating Lung Mass, Interstitial Lung Disease, Lung Disease, Lung Nodule, Obesity Hypoventilation Syndrome, Obstructive Sleep Apnea, Oxygen Dependence, Pulmonary Contusion, Pulmonary Hypertension, Recurrent Aspiration, Recurrent Aspiration Pneumonia, Recurrent Pulmonary Nodular Amyloidosis, Restrictive Lung Disease, Sleep Apnea, Tuberculosis, and Latent Tuberculosis
Chronic Kidney Disease	Chronic Kidney Disease, End Stage Renal Disease, and Renal Insufficiency

Table S2. Characteristics of the cohort based on ATSG outcomes in 824 hospitalized patients with COVID-19.

Demographics	Overall (*n* = 824)	ATSG non-severe (*n* = 545, 66.1%)	ATSG severe (*n* = 279, 33.9%)	*p*-Value [+]
**Age on Admission**	63 (IQR: 51–75) *	61 (IQR: 49–73)	66 (IQR: 57–79)	<0.001
**LOS (days)**	8 (IQR: 4–12)	6 (IQR: 4–10)	12 (IQR: 7–19)	<0.001
Number of patients with LOS ≥ 5 days	587 (71.2%) **	345 (63.3%)	242 (86.7)	<0.001
**Sex**				0.478
Female	314 (38.1%)	203 (37.2%)	111 (39.8%)	
Male	510 (61.9%)	342 (62.8%)	168 (60.2%)	
**Ethnicity**				0.009
African American	115 (14.0%)	81 (15.0%)	33 (11.8%)	
Asian	57 (6.8%)	35 (6.4%)	22 (7.9%)	
Hispanic	276 (33.5%)	203 (37.2%)	73 (26.2%)	

Demographics	Overall (*n* = 824)	ATSG non-severe (*n* = 545, 66.1%)	ATSG severe (*n* = 279, 33.9%)	*p*-Value +
Other	26 (3.2%)	16 (2.9%)	10 (3.6%)	
South Asian	46 (5.6%)	28 (5.1%)	18 (6.5%)	
White	304 (36.9%)	181 (33.2%)	123 (44.1%)	
**BMI Group**				0.126
Underweight or Normal	262 (31.8%)	164 (30.1%)	98 (35.1%)	
Overweight	276 (33.5%)	195 (35.8%)	81 (29.0%)	
Obese	286 (34.7%)	186 (34.1%)	100 (35.8%)	
**Comorbidity**				
Obesity	288 (35.0%)	187 (34.3%)	101 (36.2%)	0.591
Hypertension	523 (63.5%)	340 (62.4%)	183 (65.6%)	0.366
CAD or MI	161 (19.5%)	91 (16.7%)	70 (25.1%)	0.004
Diabetes	330 (40.0%)	207 (38.0%)	123 (44.1%)	0.091
Chronic Kidney Disease	91 (11.0%)	52 (9.5%)	39 (14.0%)	0.054
Chronic Liver Disease	23 (2.8%)	9 (1.7%)	14 (5.0%)	0.005
Autoimmune Condition	41 (5.0%)	23 (4.2%)	18 (6.5%)	0.163
Asthma	69 (8.4%)	49 (9.0%)	20 (7.2%)	0.371
COPD	71 (8.6%)	44 (8.1%)	27 (9.7%)	0.437
Pulmonary - Other	55 (6.7%)	33 (6.1%)	22 (7.9%)	0.319
Malignancy (history of)	110 (13.3%)	64 (11.7%)	46 (16.5%)	0.058
**Clinical Outcome**				<0.001
Survived	641 (77.8%)	545 (100%)	96 (34.4%)	
Died	183 (22.2%)	0 (0%)	183 (65.6%)	

LOS: length of stay; CAD: coronary artery disease; MI: myocardial infarction; COPD: chronic obstructive pulmonary disease. For binning methods of comorbidities refer to Table S1.
* Median (IQR) for continuous variables.
** Counts (percent within the group) for categorical variables.
+ Statistical tests used to compare ATSG severe and non-severe groups: Wilcoxon rank-sum test for continuous variables, chi-square test without Yates's correction for categorical variables.

Table S3. Results of laboratory tests obtained early in the hospitalization of 824 COVID-19 patients in relation to ATSG outcomes.

Laboratory Values *[Median (IQR)]*	Overall (*n* = 824)	ATSG non-severe (*n* = 545, 66.1%)	ATSG severe (*n* = 279, 33.9%)	*p*-Value *	Reference Range
**C-Reactive Protein**					
Max CRP	15.02 (7.71–25.24)	12.07 (6.20–21.07)	22.00 (12.74–31.13)	<0.001	< 0.07 mg/dL
On Admission	10.32 (5.26–17.63)	8.97 (4.61–15.87)	12.87 (7.00–21.62)	<0.001	< 0.07 mg/dL
**Admission values**					
[Eosinophil]	0.00 (0.00–0.03)	0.01 (0.00–0.03)	0.00 (0.00–0.01)	<0.001	0.03–0.27 x $10^3/\mu L$
[Lymphocyte]	0.83 (0.59–1.21)	0.88 (0.64–1.26)	0.77 (0.53–1.11)	<0.001	1.16–3.18 x $10^3/\mu L$
[Neutrophil]	6.10 (4.34–8.83)	5.85 (4.17–8.02)	6.85 (4.76–10.08)	<0.001	2.00–7.15 x $10^3/\mu L$
BUN	18 (11–32)	15 (11–25)	24 (15–42)	<0.001	6–23 mg/dL
D-Dimer	1091 (636–1932)	964 (560–1708)	1199 (832–2501)	<0.001	0–500 ng/mL
Ferritin	779 (410–1475)	728 (384–1334)	933 (448–1697)	0.004	20–335 ng/mL
INR	1.17 (1.08–1.29)	1.16 (1.08–1.27)	1.19 (1.09–1.32)	0.059	0.8–1.1
[WBC]	7.9 (5.9–10.7)	7.6 (5.7–9.9)	8.4 (6.2–12.3)	<0.001	4.0–10.0 x $10^3/\mu L$

Max CRP: maximum CRP value within 5 days of admission; [] indicate absolute cell counts. * Wilcoxon rank-sum test used to compare ATSG severe and non-severe groups.

Table S4. Results of linear regression and logistic regression models of sex, BMI, age, and CRP in relation to ATSG outcomes.

Regression Models	Odds Ratio (95% CI)	*p*-Value
**CRP**		
Initial CRP (continuous) → ATSG	1.009 (1.006, 1.012)	<0.001
Initial CRP (>10 mg/dL) → ATSG	1.80 (1.34, 2.42)	<0.001
MAX CRP (continuous) → ATSG	1.012 (1.010, 1.015)	<0.001
MAX CRP (>10 mg/dL) → ATSG	3.38 (2.39, 4.87)	<0.001
**Age**		
Age (≥60 years old) → ATSG	1.93 (1.42, 2.62)	<0.001
Age (≥60 years old) → MAX CRP (>10 mg/dL)	0.93 (0.70, 1.25)	0.65
Age (≥60 years old) × MAX CRP (>10 mg/dL) → ATSG *	1.17 (0.53, 2.47)	0.69
**Sex**		
Sex (M vs. F) → ATSG	0.90 (0.67, 1.21)	0.48
Sex (M vs. F) → MAX CRP (>10 mg/dL)	1.44 (1.07, 1.94)	0.015
Sex (M vs. F) × MAX CRP (>10 mg/dL) → ATSG	1.28 (0.62, 2.63)	0.50
Sex (M vs. F) × Age (≥60 years old) → ATSG	1.18 (0.61, 2.25)	0.63
**BMI**		
BMI (≥25 kg/m^2) → ATSG	0.80 (0.59, 1.08)	0.14
BMI (≥25 kg/m^2) → MAX CRP (>10 mg/dL)	1.65 (1.21, 2.24)	0.0014
BMI (≥25 kg/m^2) × MAX CRP (>10 mg/dL) → ATSG	1.07 (0.51, 2.22)	0.85
BMI (≥25 kg/m^2) × Age (≥60 years old) → ATSG	0.52 (0.25, 1.06)	0.078

* Two-way multivariate logistic regression models are symbolized by "×" between the two variables.

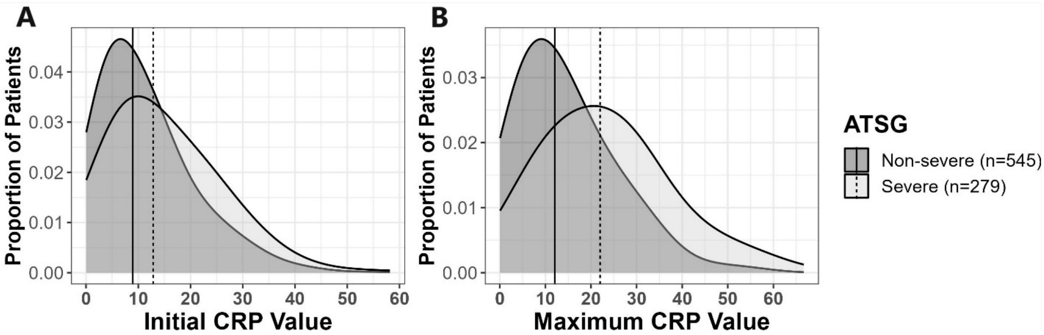

Figure S1. Distribution of initial and maximal CRP values based on ATSG classification. For each graph, the dashed black line represents the median of the corresponding CRP values among ATSG severe patients, and the solid black lines represent the median of the corresponding CRP values among ATSG non-severe patients. Panels: (**A**) distribution of initial CRP. For ATSG severe patients, the median initial CRP was 12.87 (IQR: 7.00–21.62) vs. 8.97 (IQR: 4.61–15.87) mg/dL for ATSG non-severe patients ($p < 0.001$); (**B**) distribution of maximum CRP. For ATSG severe patients, the median MAX CRP was 22.00 (IQR: 12.74–31.13) vs. 12.07 (IQR: 6.20–21.07) mg/dL for ATSG non-severe patients ($p < 0.001$).

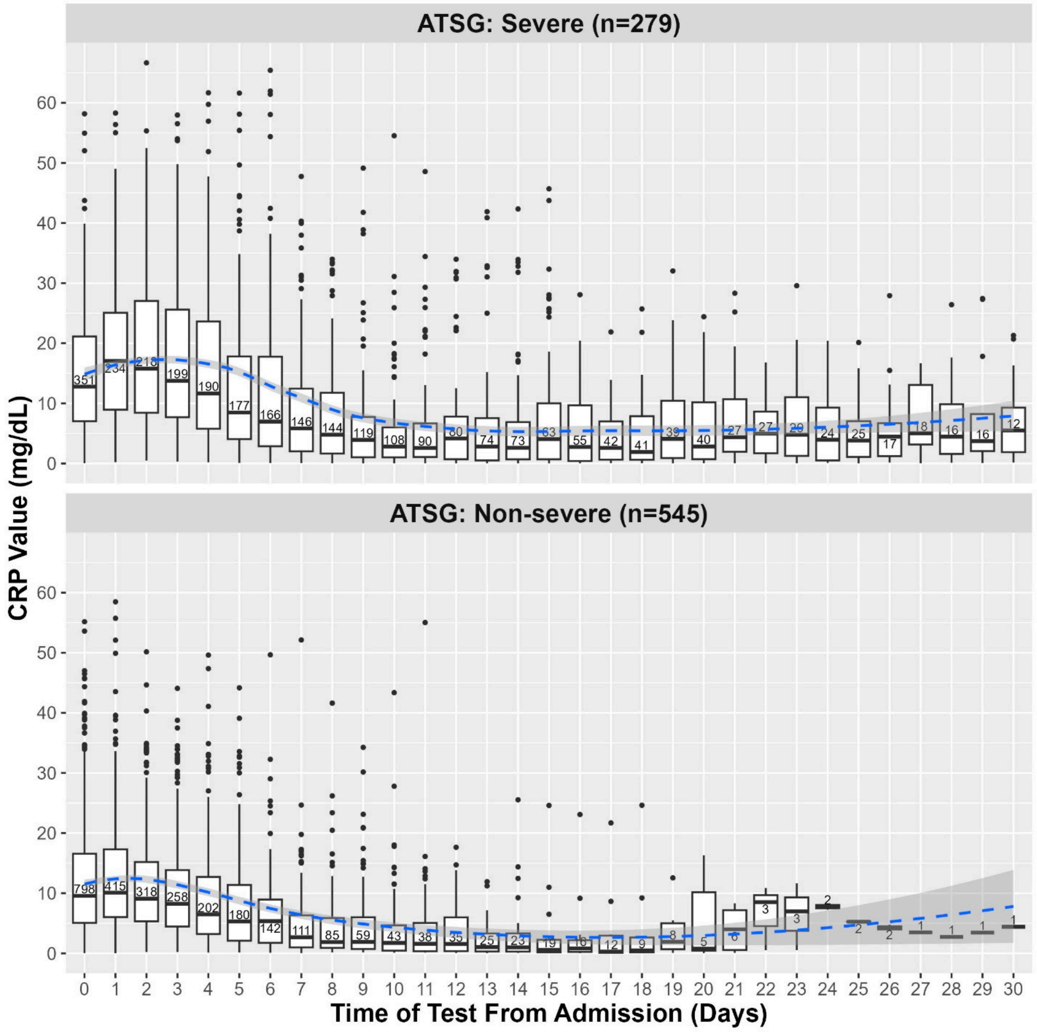

Figure S2. CRP levels within the first 30 days of hospital admission based on ATSG classification. The values shown on day 0 are defined by CRP values collected within the 24h preceding hospital admission. Each boxplot shows the median and IQR of CRP values; points are outliers, and in each box is written the number of CRP values obtained on the corresponding day. The blue line represents a local regression plotting CRP values over time, and the shaded area corresponds to the 95% confidence interval of the regression.

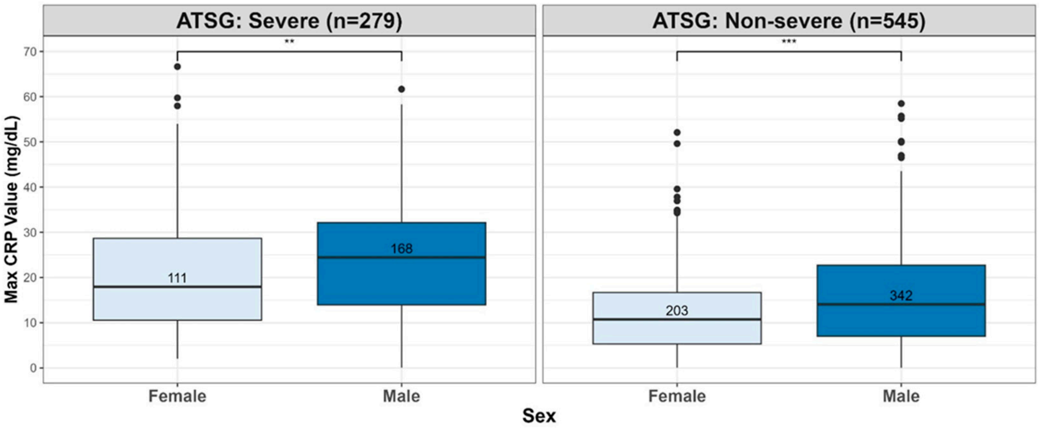

Figure S3. Maximum CRP values based on sex and ATSG classification. Each boxplot shows median and IQR of MAX CRP values, points are outliers, and in each box is written the number of patients within the corresponding patient group. Statistical significance is determined by Wilcoxon rank sum test. Males had significantly higher MAX CRP levels than females among both ATSG severe patients

(24.45 [IQR: 13.96–32.15] vs. 17.96 [IQR: 10.55–28.68] mg/dL; p = 0.004) and ATSG non-severe patients (14.04 [IQR: 7.00–22.74] vs. 10.75 [IQR: 5.30–16.69] mg/dL; p < 0.001). ** p < 0.01. *** p < 0.001.

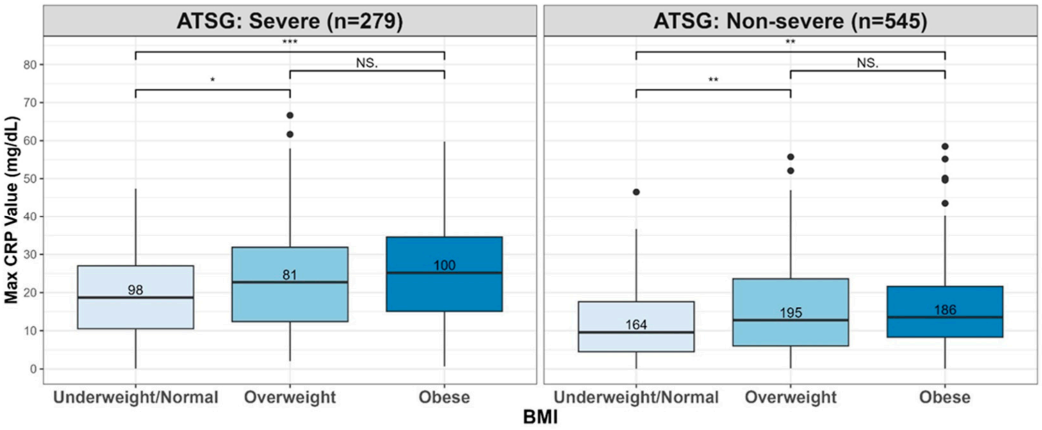

Figure S4. Maximum CRP values based on BMI tertiles and ATSG classification. Each boxplot shows median and IQR of MAX CRP values, points are outliers, and in each box is written the number of patients within the corresponding patient group. Statistical significance is determined by Wilcoxon rank sum test. Obese patients had significantly higher MAX CRP levels than underweight / normal weight patients among both ATSG severe patients (25.18 [IQR: 15.07–34.53] vs. 18.70 [IQR: 10.50–26.99] mg/dL; p < 0.001) and ATSG non-severe patients (13.54 [IQR: 8.31–21.60] vs. 9.61 [IQR: 4.49–17.60] mg/dL; p = 0.001). Overweight patients had significantly higher MAX CRP levels than underweight / normal weight patients among both ATSG severe patients (22.71 [IQR: 12.39–31.85] vs. 18.70 [IQR: 10.50–26.99] mg/dL; p = 0.024), and ATSG non-severe patients (12.78 [IQR: 6.04–23.59] vs. 9.61 [IQR: 4.49–17.60] mg/dL; p = 0.007). Obese patients did not have significantly higher MAX CRP levels than overweight patients among either ATSG severe patients (25.18 [IQR: 15.07–34.53] vs. 22.71 [IQR: 12.39–31.85] mg/dL; p = 0.327) or ATSG non-severe patients (13.54 [IQR: 8.31–21.60] vs. 12.78 [IQR: 6.04–23.59] mg/dL; p = 0.602). NS. No Significance. *p<0.05. **p<0.01. ***p<0.001.

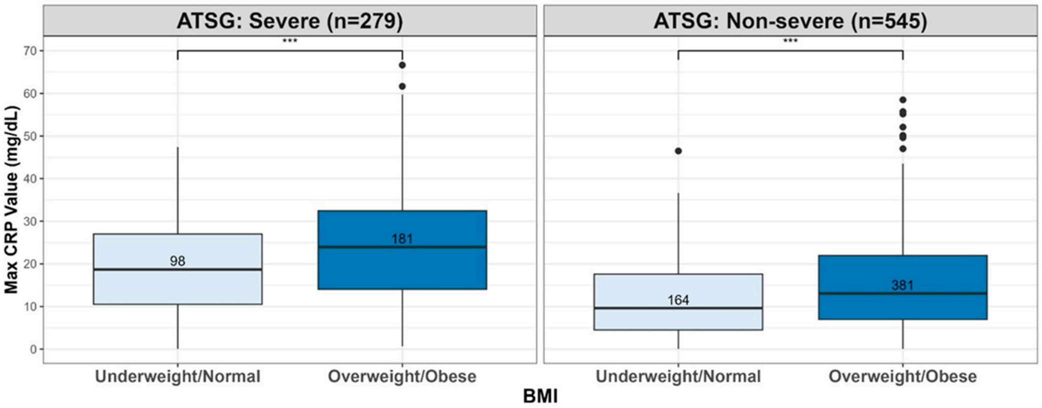

Figure S5. Maximum CRP values based on BMI and ATSG classification. Each boxplot shows median and IQR of MAX CRP values, points are outliers, and in each box is written the number of patients within the corresponding patient group. Statistical significance is determined by Wilcoxon rank sum test. Overweight/obese patients had significantly higher MAX CRP levels than underweight / normal weight patients among both ATSG severe patients (23.95 [IQR: 14.06–32.47] vs. 18.70 [IQR: 10.50–26.99] mg/dL; p < 0.001) and ATSG non-severe patients (13.05 [IQR: 6.99–21.98] vs. 9.61 [IQR: 4.49–17.60] mg/dL; p < 0.001). ***p<0.001.

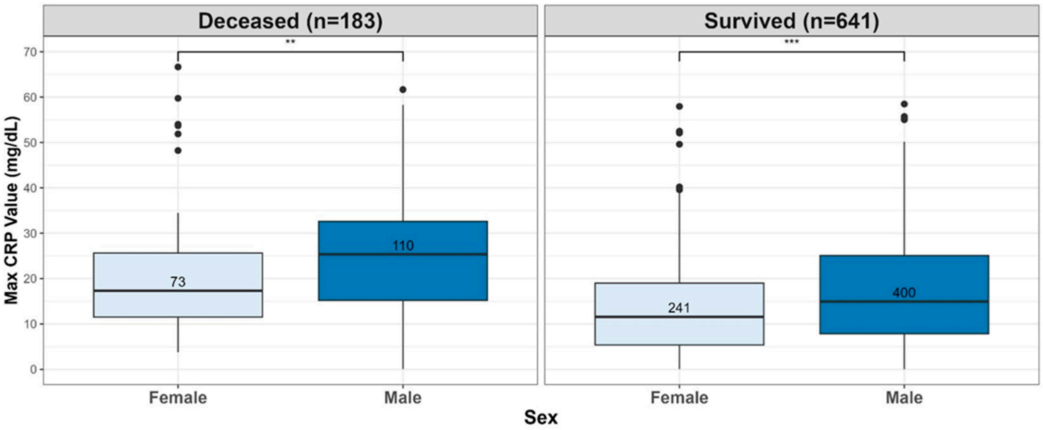

Figure S6. Maximum CRP values based on sex among survivors and deceased. Each boxplot shows median and IQR of MAX CRP values, points are outliers, and in each box is written the number of patients within the corresponding patient group. Statistical significance is determined by Wilcoxon rank sum test. Males had significantly higher MAX CRP levels than females among both deceased (25.37 [IQR: 15.22–32.62] vs. 17.31 [IQR: 11.51–25.64] mg/dL; p = 0.002) and survivors (14.96 [IQR: 7.84–25.08] vs. 11.59 [IQR: 5.39–19.00] mg/dL; p < 0.001). ** p < 0.01. *** p < 0.001.

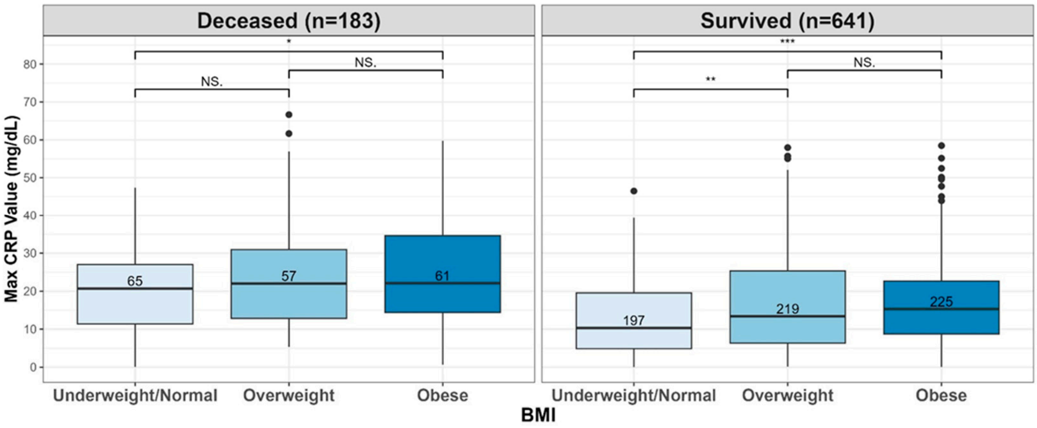

Figure S7. Maximum CRP values based on BMI tertiles among survivors and deceased. Each boxplot shows median and IQR of MAX CRP values, points are outliers, and in each box is written the number of patients within the corresponding patient group. Statistical significance is determined by Wilcoxon rank sum test. Obese patients had significantly higher MAX CRP levels than underweight / normal weight patients among both deceased (22.09 [IQR: 14.38–34.62] vs. 20.70 [IQR: 11.37–27.03] mg/dL; p = 0.020) and survivors (15.31 [IQR: 8.76–22.67] vs. 10.31 [IQR: 4.89–19.54] mg/dL; p < 0.001). Overweight patients had significantly higher MAX CRP levels than underweight / normal weight patients among survivors (13.38 [IQR: 6.32–25.33] vs. 10.31 [IQR: 4.89–19.54] mg/dL; p = 0.006), but did not have significantly higher MAX CRP levels than underweight / normal weight patients among deceased (22.01 [IQR: 12.83–30.95] vs. 20.70 [IQR: 11.37–27.03] mg/dL; p = 0.154). Obese patients did not have significantly higher MAX CRP levels than overweight patients among either deceased (22.09 [IQR: 14.38–34.62] vs. 22.01 [IQR: 12.83–30.95] mg/dL; p = 0.443) or survivors (15.31 [IQR: 8.76–22.67] vs. 13.38 [IQR: 6.32–25.33] mg/dL; p = 0.269). NS. No Significance. * p < 0.05. ** p < 0.01. *** p < 0.001.

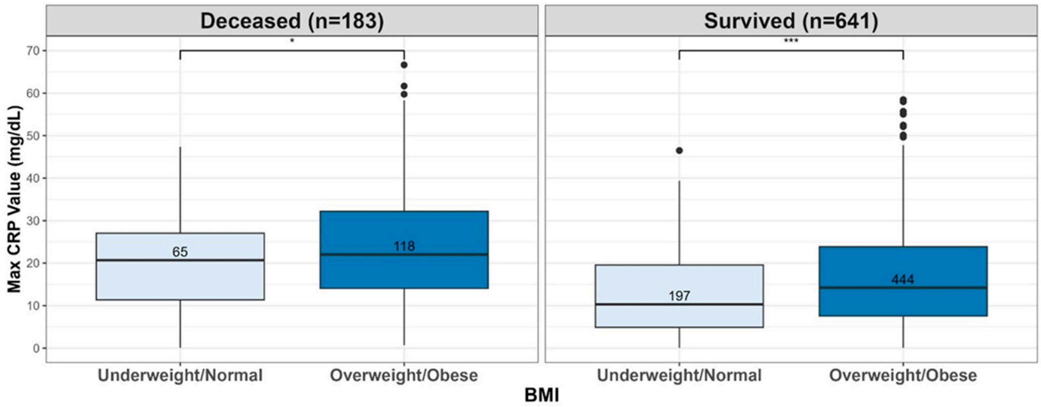

Figure S8. Maximum CRP values based on BMI among survivors and deceased. Each boxplot shows median and IQR of MAX CRP values, points are outliers, and in each box is written the number of patients within the corresponding patient group. Statistical significance is determined by Wilcoxon rank sum test. Overweight/obese patients had significantly higher MAX CRP levels than underweight / normal weight patients among both deceased (22.05 [IQR: 14.08–32.21] vs. 20.70 [IQR: 11.37–27.03] mg/dL; $p = 0.028$) and survivors (14.24 [IQR: 7.55–23.87] vs. 10.31 [IQR: 4.89–19.54] mg/dL; $p < 0.001$). *$p<0.05$. ***$p<0.001$.
